# Aristolochic Acid Affects Upper Tract Urothelial Cancer Behavior through the MAPK Pathway

**DOI:** 10.3390/molecules24203707

**Published:** 2019-10-15

**Authors:** I-Hsuan Chen, Hao-Lun Luo, Yu-Li Su, Chun-Chieh Huang, Po-Hui Chiang, Chia-Cheng Yu, Nai-Lun Lee, Jen-Jie Lin, Ming-Tse Sung

**Affiliations:** 1 Division of Urology, Department of Surgery, Kaohsiung Veterans General Hospital, Kaohsiung 81362, Taiwan; alan_aries@yahoo.com.tw (I.-H.C.); mlee0857@gmail.com (C.-C.Y.); 2 Division of Urology, Department of Surgery, Tri-Service General Hospital, National Defense Medical Center, Taipei 11490, Taiwan; 3 National Yang-Ming University, School of Medicine, Taipei 11221, Taiwan; 4 Department of Urology, Kaohsiung Chang Gung Memorial Hospital and Chang Gung University College of Medicine, Kaohsiung 83301, Taiwan; alesy1980@gmail.com (H.-L.L.); tuoa480713@gmail.com (P.-H.C.); nailun0110@gmail.com (N.-L.L.); 5 Department of Hematology and Oncology, Kaohsiung Chang Gung Memorial Hospital and Chang Gung University College of Medicine, Kaohsiung 83301, Taiwan; yolisu@mac.com; 6 Department of Radiation Oncology, Kaohsiung Chang Gung Memorial Hospital and Chang Gung University College of Medicine, Kaohsiung 83301, Taiwan; cgukinace@gmail.com; 7 Department of Pharmacy, College of Pharmacy and Health Care, Tajen University, Pingtung 90741, Taiwan; 8 Department of Beauty Science, Meiho University, Pingtung 91202, Taiwan; 9 Department of Pathology, Kaohsiung Chang Gung Memorial Hospital and Chang Gung University College of Medicine, Kaohsiung 83301, Taiwan

**Keywords:** aristolochic acid, UTUC, tumorigenic, MAPK pathway

## Abstract

The prevalence of upper tract urothelial carcinoma (UTUC) in Taiwan is relatively higher than thatin Western countries. Aristolochic acid (AA), which is widely used in traditional Chinese herbology, is now recognized to be one of the carcinogens for UTUC. Numerous UTUC patients have chronic kidney diseases or end-stage renal diseases; however, little literature hasreported on theoncogenic pathway of AA-related UTUC. The aim of our study was to identify the potential target treatment for AA-related UTUC. Here, we established an AA pre-exposure followed bya 3-methylcholanthrene (MCA) stimulus tumorigenic cell model. We not only demonstrated that AA pre-exposure MCA stimulus tumorigenic cells have more behaviors of cell migration and invasion by enhancing the metalloproteinases (MMP) activity, which is compatible with clinical findings of AA-related UTUC, but we also validated that AA pre-exposure MCA stimulus tumorigeniccells could be activated through the mitogen-activated protein kinases (MAPK) pathway. We further dissected the route of the MAPK pathway and found that the p38 and extracellular signal regulated kinases (ERK) sub-pathways might play essential roles in AA pre-exposure urothelial cancer cell lines. This consequence was also corroborated with a tissue study in AA-exposed patients.

## 1. Introduction

Over the last fewdecades, the incidence of urothelialcancers, which accounted for 10% of all new malignancies in 2012, has gradually increased in Taiwan [1,2]. Upper tract urothelial carcinoma (UTUC), arising from the urothelial lining of the urinary tract from renal calyces to the ureteral orifice, comprises only 5% of all urothelial carcinomas in Western countries [3,4,5], but it represents 31% of all urothelial carcinomas in Taiwan [6,7,8]. The high prevalence of UTUC has identified Taiwan as an endemic area [9].

Aristolochic acid (AA), which is widely prescribed in traditional Chinese herbal medicine, was first reported to be associated with nephrotoxicity in the 1990s [10,11]. A group of healthy Belgian women developed renal failure after taking AA-containing herbs under a weight-loss regimen [12]. Nowadays, AA is recognized as a human carcinogen with a high correlation with the development of UTUC. It is worthnoting that AA-induced UTUC (AA-UTUC) patients tend to be younger and have poorer renal function, contributing to difficulties in cancer treatment [13,14,15]. About 26% and 40% of patients with UTUC have beenreported to have AA-UTUC or possibly have AA-UTUC, respectively, in Taiwan [13]. For those with AA-UTUC, synchronous and metachronous diseases are common. Therefore, the risk of disease progression and repeated surgical intervention cancause shortened survival or increased disability [16].

With regards to the aggressive behavior of AA-UTUC, biomarkers such as the mutational signature associated with AA (A:T to T:A transversion in TP53) and DNA-AA adducts in the renal cortex have been identified as being related [17,18]. Research into related cancer pathways may help to improve treatment modality in such patients. Accordingly, in our study, an AA-exposure normal urothelial cell model was designed to investigate whether the MAPK pathway could be a possible carcinogenic mechanism. The results were then verified via a western blot analysis of extracts from UTUC patients with or without AA exposure.

## 2. Results

### 2.1. Cytotoxicity of Aristolochic Acid (AA) in SV-HUC-1 Cells and the Effect of a Low Concentration of AA on MCA-Induced Tumorigenic Transformation

Many studies have confirmed that AA, used in Chinese herbal medicine, may cause renal and urinary tract epithelial cancer and other tissue diseases. Therefore, we established an in vitro model of the long-term exposure of cells to AA, and we further explored the molecular mechanism associated with tumorigenic transformation. 

**Figure 1:**
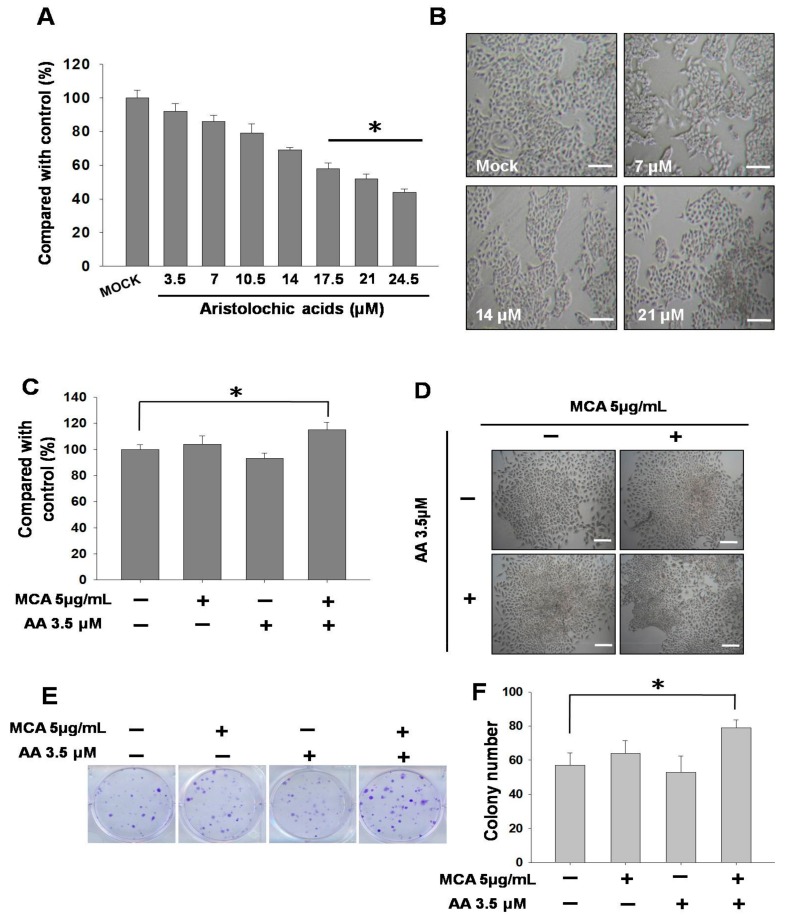
Effects of aristolochic acid (AA) on inhibition and tumorigenic transformation in SV-HUC-1 cells. Data are presented as mean ± SEM from three independent experiments. (**A**) Viability of SV-HUC-1 cells at different concentrations of AA (0–24.5 μM) incubation for 24 h. Inhibitory effects on cell proliferation were assessed by a cytotoxicity assay as described in Materials and Methods. (**B**) Cell morphology of SV-HUC-1 cells treated with different concentrations of AA (0, 7, 14, and 21 μM) incubation for 24 h. (**C**) Viability of SV-HUC-1 cells treated with AA (3.5 μM) and 3-methylcholanthrene (MCA) (5 μg/mL) incubation for 24 h.Inhibitory effects on cell proliferation were assessed by a cytotoxicity assay as described in Materials and Methods. (**D**) Cell morphology of SV-HUC-1 cells treated with AA (3.5 μM) and MCA (5 μg/mL) incubation for 24 h. (**E**) Clonogenic assay of cells treated with AA (3.5 μM) and MCA (5 μg/mL) for 13 days. (**F**) Quantification results of the clonogenic assay. Scale bar = 20 μm. # *p* < 0.05, * *p* < 0.001.

First, we first tested the toxicity of AA towards SV-HUC-1 cells. The results showed that with an increasing AA concentration (0–24.5 μM), the survival rate of SV-HUC-1 cells gradually decreased, demonstrating that AA induced cytotoxicity in the cells. An analysis of cell numbers under treatment with different concentrations of AA (mock, 7, 14, and 21 μM) showed that an approximate 30% reduction was seen in cell numbers under AA treatment at a concentration of 14 μM (Figure 1A). An assessment of cell modality using a microscope demonstrated that AA caused cell-membrane shrinkagerelated to a decrease of cytoplasmand changes in cell density (Figure 1B), and this result was consistent with that shown in Figure 1A.

We subsequently used AA at a lower toxicity (3.5 μM) for the treatment of SV-HUC-1 cells, followed by the addition of 3-methylcholanthrene (MCA) to induce tumorigenic transformation. The results showed that when only MCA (5 μg/mL) was administered, the stimulation led to an increase in the number of cells; however, the administration of MCA after AA treatment further increased cell growth (Figure 1C). In terms of cell morphology, it was found that the cell density of SV-HUC-1 cells after long-term AA treatment was higher (Figure 1D). In order to verify this result, we used a clonogenic assay to investigate whether the proliferative capacity of the cells was increased. The quantitative results confirmed significant differences and demonstrated that there were more colonies in cells treated with AA and MCA (Figure 1E,F).

### 2.2. Changes in Cell Behavior and Matrix Metalloproteinase after Exposure to Aristolochic Acid

**Figure 2:**
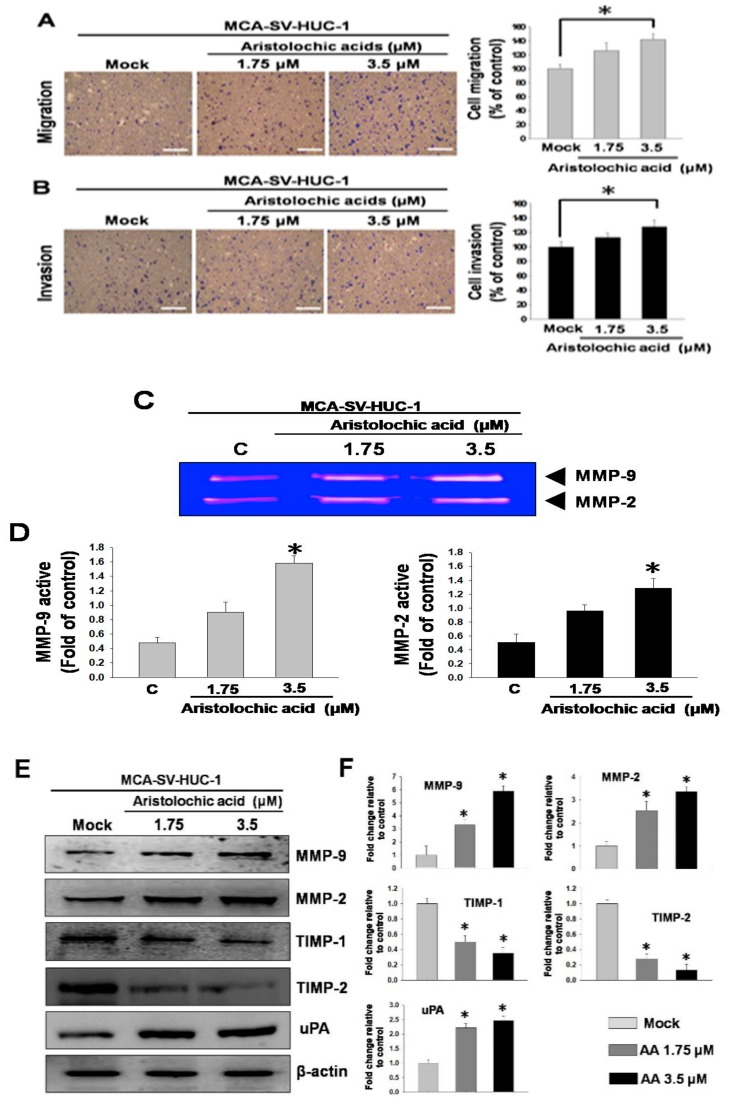
Aristolochic acid (AA) promoted cell migration and invasion in MCA-SV-HUC-1 cells. Data are presented as mean ± SEM from three independent experiments. (**A**) Effect of AA (0, 1.75, 3.5 μM) on cell migration. (**B**) Effect of AA on cell invasion. (**C**) Gelatin zymography of metalloproteinase-2 (MMP-2) and MMP-9 activities in MCA-SV-HUC-1 cells treated with AA. (**D**) Quantification of MMP-9 and MMP-2 zymograms. (**E**) Western blotting of changes in MMP-2, MMP-9, tissue inhibitor metalloproteinase-1 (TIMP-1), TIMP-2 and urokinase-type plasminogen activator (uPA) levels in MCA-SV-HUC-1 cells treated with AA. (**F**) Quantification of protein concentrations using Image J 1.47 software (National Institutes of Health, Bethesda, MD, USA). Scale bar = 20 μm. # *p* < 0.05, * *p* < 0.001.

Subsequently, we investigated whether exposure to AA affected cell behavior. Transwell migration and invasion assays were performed to simulate the cell movement caused by AA treatment, and the results showed that the MCA-induced cell migration and invasion abilities in MCA-SV-HUC-1 cells were increased with increasing AA concentrations (Figure 2A,B), indicating that AA is associated with increases in cell motility and invasiveness. The results suggested that AA can cause an increase in metastatic capacity.

We further explored the underlying molecular mechanism related to the aforementioned results. During cell migration, cells need to decompose the extracellular matrix by expressing matrix metalloproteinases (MMPs). Therefore, an increase in the capacity for neoplastic transformation is normally correlated with augmented MMP activities in the cells. MMP zymography showed that the enzyme activities of MMP-2 and MMP-9 were significantly higher with the application of increasing AA concentrations in MCA-SV-HUC-1 cells (Figure 2C,D), thus indicating that exposure to AA resulted in the overexpression of MMP-2 and MMP-9 in the cells. Additionally, a western blot analysis demonstrated that the levels of MMP-2, MMP-9 and urokinase-type plasminogen activator (uPA) were increased, and the levels of tissue inhibitor metalloproteinase-1 (TIMP-1) and TIMP-2 were reduced (Figure 2E). These results showed that enzyme activities and protein levels in the cells, which contribute substantially to increased cell migration and invasion, were increased under AA treatment.

### 2.3. Aristolochic Acid Induces Cell Migration via Signal Transduction of ERK and p38 MAPK

**Figure 3:**
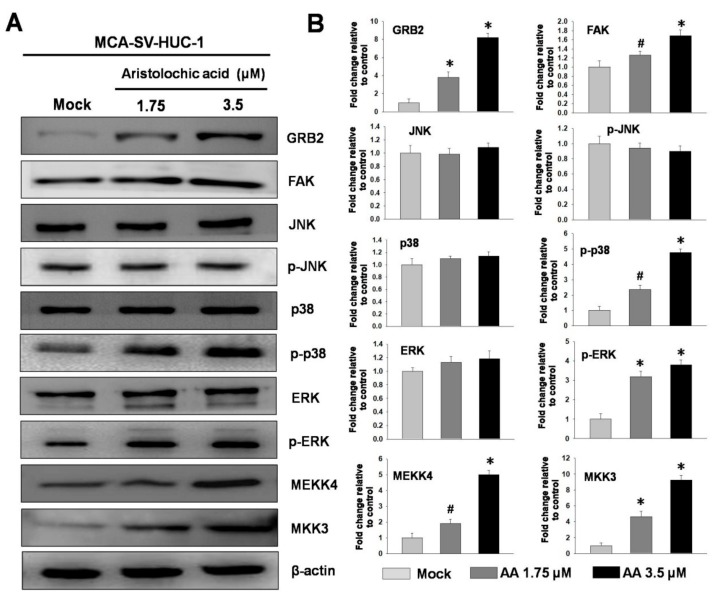
Effect ofdifferent concentrations aristolochic acid (AA) on expressions of MAPK proteins in MCA-SV-HUC-1 cells. (**A**) Determined by western blotting. (**B**) Quantification of protein concentrations using Image J 1.47 software (National Institutes of Health, Bethesda, MD, USA). # *p* < 0.05, * *p* < 0.001.

Previous studies have indicated that the expression of MMPs can be regulated by the MAPK pathway. Metastasis and invasion processes in human cells require the activation of the MAPK signaling pathway [19,20]; therefore, we used protein immunostaining to study MCA-SV-HUC-1 treated with the different concentrations of AA (mock, 1.75, and 3.5 μM) to see theeffect of AA on the MAPK signaling pathway. The results showed that the higher the concentration of AA, the greater the phosphorylation of p38 and extracellular signal regulated kinases (ERK), but no significant change was observed for c-jun N-terminal kinase (JNK), while the upstream of MAPK, growth factor receptor-bound protein 2 (GRB2) and focal adhesion kinase (FAK) were increased, and MAPK phosphorylation kinases MEKK4 (MAPK kinase kinasekinase4) and mitogen-activated protein kinase kinase3 (MKK3) also showed significant upward trends, indicating that AA may pass through the p38 and ERK pathways to induce MCA-SV-HUC-1 cell metastasis (Figure 3).

### 2.4. MAPK Inhibitors Block Aristolochic Acid-Induced Cell Migration through the ERK and p38 MAPK Pathways

**Figure 4:**
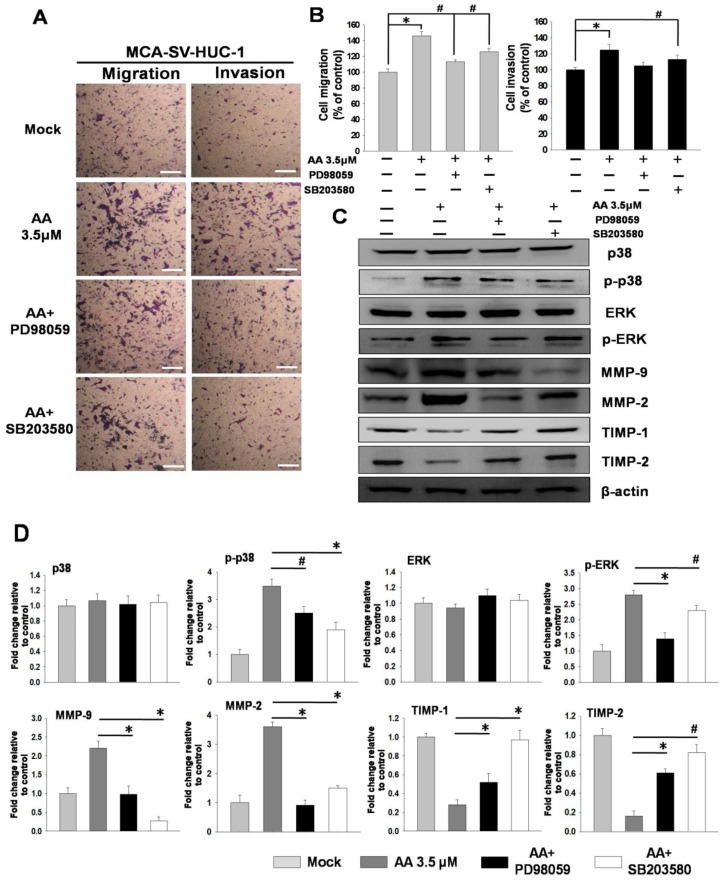
MAPK inhibitors blocked the aristolochic acid (AA)-induced signaling pathway in MCA-SV-HUC-1 cells. The detail was described in Methods Section 4.2.3. (**A**) Effects of extracellular signal regulated kinases (ERK) and p38 inhibitors on cell migration and invasion. (**B**) Quantification of cell migration and invasion. (**C**) Changes of MMP-2, MMP-9, TIMP-1, TIMP-2 and uPA in MCA-SV-HUC-1 cells treated with ERK and p38 inhibitors, as determined by western blotting. (**D**) Quantification of protein concentrations using Image J 1.47 software (National Institutes of Health, Bethesda, MD, USA). Scale bar = 20 μm. # *p* < 0.05, * *p* < 0.001.

Previous studies have found that AA may induce bladder cell migration and invasion through the ERK and p38 MAPK pathways. Therefore, we pretreated MCA-SV-HUC-1 cells with an ERK inhibitor (PD98059, 2 μM) and a p38 MAPK inhibitor (SB203580, 20 μM) for 2 h, followed by incubation with AA for 24 h. The results showed that the pretreatment of MCA-SV-HUC-1 cells with PD98059 and SB203580 followed by AA treatment inhibited cell migration and invasion. Moreover, the protein levels of MMP-2 and MMP-9 were reduced, while the expressions of TIMP-1 and TIMP-2 were increased (Figure 4).

### 2.5. Expression of MAPK Proteins in UTUC tissues of Patients Exposed to Aristolochic Acid

**Figure 5:**
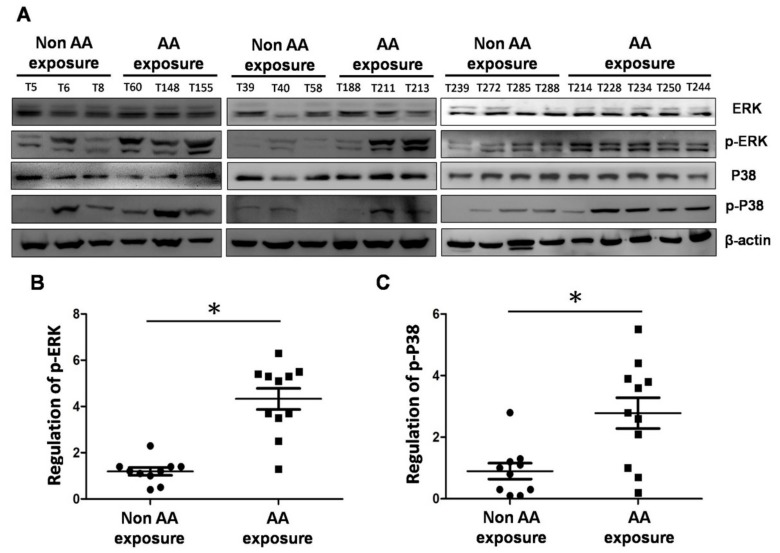
Comparison of expressions of ERK, p38, phosphorylated extracellular signal regulated kinases (p-ERK) and p-p38 proteins in tissues of patients with exposure to AA and those without. (**A**) Western blotting of ERK, p38, p-ERK and p-p38 expressions in the tissues of patients with or without exposure to AA. (**B**) Quantification of p-ERK protein expression. (**C**) Quantification of p-p38 protein expression. # *p* < 0.05, * *p* < 0.001.

The results of the in vitro experiments described above suggested that cells with prolonged exposure to AA followed by contact with a carcinogen overexpress phosphorylated ERK and p38 MAPK, which may induce increases in neoplastic transformation and metastasis. Therefore, we collected UTUC tissue samples from patients who had engaged inthe long-term use of traditional Chinese herbal medicine (N = 11) and who were likely have been exposed to AA, and we compared these with tissues of subjects who did not take traditional Chinese herbal medicine (N = 10) in terms of the expressions of MAPK proteins, including phosphorylated extracellular signal regulated kinases (p-ERK) and p-p38, by western blotting. Of the patients with AA exposure, nine (9/11, 81.8%) had p-ERK overexpression and eight (8/11, 72.7%) had p-p38 overexpression in their tissues (Figure 5). This in vivo finding suggested that exposure to AA can cause the overexpression of p-ERK and p-p38, which in turn may lead to urinary and kidney diseases.

## 3. Discussion

Previous studies have reported that AA, which is present in some traditional Chinese herbal medicines, shows high correlations with UTUC and chronic kidney disease in Taiwan [21,22]. In addition, some researchers have pointed out that AA is associated with increased risks of end-stage renal disease and urothelial cancer [23,24], and, in these types of cancer, up to 43% are located in the upper urinary tract [25]. Many patients with UTUC have chronic kidney disease or end-stage renal disease [26], which results in a limited choice of chemotherapy in the case of advanced malignancies [27,28]. If chemotherapy is not performed, the prognosis of advanced UTUC is usually unsatisfactory. Therefore, AA plays a key role in UTUC.

Thus, this study investigated the tumorigenic transformation process of urinary epithelial cells induced by prolonged exposure to AA and explored the underlying mechanisms of such. We used the human normal urinary epithelial SV-HUC-1 cell line as an in vitro model and demonstrated that AA exposure may promote cell proliferation in urinary epithelial cells. Though higher concentrations of AA resulted in cytotoxicity in SV-HUC-1 cells, long-term treatment with a lower dose of AA increased cell proliferation, with a denser cell-growth pattern in a clonogenic assay, when the cells subsequently underwent MCA-induced tumorigenic transformation. The results confirmed that AA may accelerate tumor cell proliferation at low concentrations under continuing use.

We then studied whether AA exposure may contribute to cell migration and invasion using transwell assays. Our results showed that both cell migration and invasion were significantly increased in cells pre-treated with a low concentration of AA (3.5 μM). Cancer cells secrete MMPs in their initial stage of invasion to break down the extracellular matrix and promote tumor-spreading into the blood vessels to other peripheral organs. Thus, the overexpression of MMPs is considered the first step in cancer-cell invasion [29]. We used zymography to analyze the effects of AA on the enzyme activities of MMPs in MCA-stimulated SV-HUC-1 cells. The results showed that cells exposed to AA for a long time exhibited increased MMP-2 and MMP-9 activities with an increasing AA concentration. Western blotting further confirmed that the protein levels of MMP-2 and MMP-9 in the cells were also increased under AA treatment, while the expressions of TIMP-1 and TIMP-2 were decreased. Additionally, uPA, a key factor in a range of events in the metastatic cascade, was also significantly increased. Our results suggested that AA indeed induces expressions of MMPs, promoting cell migration and invasion.

Previous studies have indicated that the activities of MMPs and uPA are regulated by MAPKs [30,31]. Therefore, we investigated whether long-term exposure to AA leads to any changes in MAPKs. Our results showed that with an increasing concentration of AA, GRB2 expression on the cell membrane was increased and the intracellular FAK expression increased, both of which further affected MAPKs. We also found that AA induced the expression of p-ERK and p-p38, while no significant change was observed for JNK, suggesting that AA treatment may increase the cell migration capacity by phosphorylating ERK and p38. The result involving the ERK pathway and subsequent MMP change is reasonable for an explanation about invasive cancer behavior because of its effect oncell motility via cell-matrix adhesion [32,33].

To confirm whether AA induces cell migration and invasion via the phosphorylation of ERK and p38, we used ERK and p38 inhibitors to pretreat MCA-SV-HUC-1 cells for 2 h, followed by incubation with AA for 24 h. An examination of cell behavior and molecular changes showed that the cell migration and invasion abilities of the cells pretreated with inhibitors were significantly decreased, and western blotting showed that the expressions of MMP-2 and MMP-9 were decreased, while TIMP-1 and TIMP-2 were increased. The findings suggested that AA-induced cell migration and invasion occur through the pathways of ERK and p38. We then collected tissues from patients who had a history of the regular intake of traditional Chinese herbal medicine and patients who rarely used traditional Chinese herbal medicines. Western blotting was performed using total proteins extracted from the tissues, and the results showed that in the patients with a history of regular intake of Chinese herbal medicine, the levels of p-ERK (9/11, 81.8%) and p-p38 (8/11, 72.7%) were increased. We concluded that AA-induced cell migration/invasion and neoplastic transformation are majorly mediated by these signal pathways, according our in vitro models. This result has been further identified in real world human UTUC samples. The potential use of targeted ERK/p38 therapy may help inhibit the metastatic process via extracellular matrix spreading in clinically AA-exposed patients.

## 4. Materials and Methods

### 4.1. Materials

Aristolochic acid (AA), 3-methylcholanthrene (MCA), 3-(4,5-dimethylthiazol- 2-yl)-2,5- diphenyltetrazolium bromide (MTT), dimethyl sulfoxide (DMSO), Coomassie Blue R-250, Ponceau S,ERK1/2 inhibitor (PD98059), p38MAPK inhibitor (SB203580), NaCl, NaN_3_, ZnCl_2_, CaCl_2_, Triton-X 100, and rabbit anti-human β-actin antibodies were obtained from Sigma (St Louis, MO, USA). Goat anti-rabbit and horseradish peroxidase-conjugated IgG and PVDF (polyvinylidenefluoride) membranes were obtained from Millipore (Bellerica, MA, USA). A chemiluminescent HRP substrate was purchased from Pierce (Rockford, IL, USA). Rabbit anti-human mitogen-activated protein kinase kinase3 (MKK3), MAPK kinase kinasekinase4 (MEKK4), focal adhesion kinase (FAK), and growth factor receptor-bound protein 2 (GRB2) antibodies were obtained from Epitomics (Burlingame, CA, USA). Rabbit anti-human TIMP-1 and TIMP-2 antibodies were obtained from ProteinTech Group (Chicago, IL, USA). Rabbit anti-human matrix metalloproteinase-2 (MMP-2), matrix metalloproteinase-9 (MMP-9), uPA, c-jun N-terminal kinase (JNK), phosphorylated c-jun N-terminal kinase (p-JNK), extracellular signal regulated kinases (ERK), phosphorylated extracellular signal regulated kinases (p-ERK), p38 and p-p38 antibodies were obtained from Cell Signaling Technology (Danvers, MA, USA).

### 4.2. Methods

#### 4.2.1. Cell Culture and Treatment with Aristolochic Acid Induced Tumorigenic Transformation

Human urinary tract epithelium SV-HUC-1cells were purchased from the Food Industry Research and Development Institute (Hsinchu, Taiwan).

SV-HUC-1 cells were cultured in Ham’s F-12 with 4 mM of L-glutamine adjusted to contain 1.5 g/L sodium bicarbonate, supplemented with 10% (*v*/*v*) FBS, 100 units/mL penicillin, 100 μg/mL of streptomycin, 1 mM of sodium pyruvate incubator with 5% CO2 at 37 °C. The induction of neoplastic transformation in SV-HUC-1 cells was achieved throughthe incubation of the cells with a fixed concentration of AA (1.75 and 3.5 μM) for 4 weeks. During that period, the culture was changed to a fresh medium with AA every three days. The aim of this procedure was to establish long-term exposure to a low concentration of AA in the cell line, followed by incubation with 3-methylcholanthrene (MCA) (5 μg/mL) for 2 weeks to induce tumorigenic transformation in SV-HUC-1 cells [34]. SV-HUC-1 were treated with AA for 4 weeks and then for another 2 weeks with MCA. We called this MCA-SV-HUC-1.

#### 4.2.2. Cytotoxicity Assay

SV-HUC-1 cells were seeded on 24-well culture plates at a density of 1 × 10^4^ cells/well. After various concentrations of aristolochic acid (mock, 3.5, 7, 10.5, 14, 17.5, 21, and 24.5 μM) incubation for 24 h, 50 μL of MTT solution (1 mg/mL in phosphate-buffered saline (PBS)) was added to each well. Cells treated with DMSO were used as blank controls. The cell culture plates were incubated at 37 °C for 4 h, and then the cells were lysed with 200 μL of DMSO. The optimal density (OD) was measured at 595 nm by a microtiter ELISA reader (Bio-Rad, Hercules, CA, USA). All the experiments were repeated three times [35].

#### 4.2.3. MCA-SV-HUC-1 Treats MAPKs Inhibitor and Culture

MCA-SV-HUC-1 cells were pretreated with an ERK1/2 inhibitor (PD98059, 5 μM) and an p38MAPK inhibitor (SB203580, 20 μM) for 2 h and then incubated with aristolochic acid (3.5 μM) for 24 h, after which cells were collected for migration, invasion and western blotting analysis [36].

#### 4.2.4. Cell Migration and Invasion Assay

MCA-SV-HUC-1 cells were seeded into a Boyden chamber (Neuro Probe, Cabin John, MD, USA) at 5 × 10^3^ cells/well in serum-free media. MCA-SV-HUC-1 cells with aristolochic acid (mock, 1.75 and 3.5 μM) were kept at 37 °C for 24 h to allow for cell migration. For the invasion assay, 10 μL Matrigel (25 mg/50 mL; BD Biosciences, MA, USA) was coated onto 8 μm pore-size polycarbonate membrane filters, and MCA-SV-HUC-1 cells were plated in the upper chamber of the Matrigel-coated transwell insert. The migrated and invaded cells on the lower chamber were fixed with 100% methanol and stained with 5% Giemsa (Merck, Germany). Cell numbers were counted using a 100× light microscope [37].

#### 4.2.5. Clonogenic Assay

SV-HUC-1 cells were seeded at 1 × 10^2^/well in a 6-well plate. AA (3.5 μM) and MCA (5 μg/mL) were added to the cells and the cultures were maintained in a 5% CO_2_ incubator at 37°C for 13 days. Thereafter, 0.1% crystal violet was added to stain the cells for 15 min, followed by washing three times with PBS. Images of the cells were then taken using a scanner and used for the subsequent analysis of cell colonies [38].

#### 4.2.6. Determination of MMP-2/MMP-9 Activities by Gelatin Zymography

MCA-SV-HUC-1 cells were treated with various concentrations of aristolochic acid (mock, 1.75 and 3.5 μM) for 24 h. To analyze the secretion of MMP-2/-9 in culture media, the collected culture media were concentrated by a speed vacuum. The samples were separated by 10% SDS-PAGE containing 0.2% gelatin under non-reducing conditions for MMP-2/-9 activity assay. The gels were washed in a wash buffer (100 mM of NaCl and 2.5% Triton X-100 in 50 mM of Tris-HCl, pH7.5) three times. Then, the gels were incubated in a reaction buffer (200 mM of NaCl, 0.02% NaN_3_, 1 μM of ZnCl_2_, 1 mM of CaCl_2_, 2% Triton-X 100 in 50 mM of Tris-HCl, pH 7.5) at 37 °C for 24 h. The gels were stained with Coomassie Blue R-250 [39].

#### 4.2.7. Protein Extraction and Immunostaining

The cell and tissue proteins were lysed in a TOOLS RIPA solution (TOOLS, Taiwan) with a protease inhibitor, The protein samples (30 μg) were separated by 12.5% SDS-PAGE and then transferred onto PVDF membrane for 1.5 h at 400 mA using Transphor TE 62 (Hoeffer), and then protein transfer was checked by staining with a Ponceau S solution. The membranes were subsequently incubated with 5% dehydrated skimmed milk in a PBS buffer (10 mM of NaH_2_PO_4_ and 130 mM of NaCl) to block nonspecific protein bindings; they werethen incubated with primary antibodies at 4 °C overnight. After a wash by PBST (PBS with 0.002% tween 80) of 5 times, the second antibodies (horseradish peroxidase conjugate goat anti-rabbit, 1:5000 in blocking solution) were added and incubated for 2 h at 4 °C [40]. The immunoreactive bands were visualized through chemiluminescence by adding ECL western blotting reagents (Pierce Biotechnology, Rockford, IL, USA).

#### 4.2.8. The Human Samples

The human UTUCsamples used in this study wereobtained from the institutional biobank in the Kaohsiung Chang Gung Medical Center. The definition of AA exposed UTUC patients wasobtained from the preoperative questionnaire and defined asthose with regular Chinese herbal medicine intake. This study was approved by IRB: 106-4117C.

#### 4.2.9. Statistical Analysis

Data analyses of the cytotoxicity assay, cell migration and invasion assays, and immunoblot protein expression were derived from three independent experiments. A Tukey–Kramer test was used for data acquisition and an analysis of variance (ANOVA), using GraphpadInstat 3 software (San Diego, CA, USA).

## 5. Conclusions

**Figure 6:**
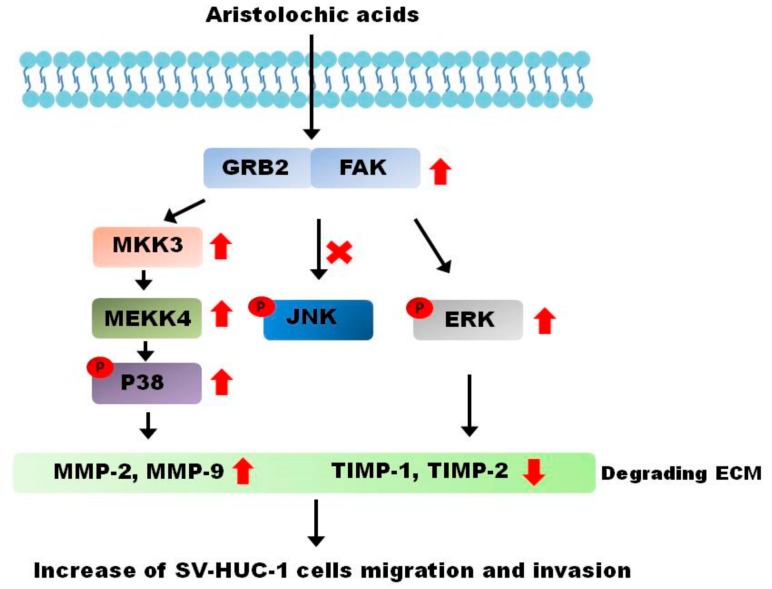
Aristolochic acid-induced cancer behavior in SV-HUC-1 cells. Based on the results of our study, the cancer behavior process caused by aristolochic acids is mediated by the ERK and p38 pathways.

We established a long-term AA-exposed upper tract urothelium model and demonstrated strongly malignant cell behavior during tumorigenic transformation following exposure to AA. Increasing expressions of MMPs, corresponding with the AA concentration, also indicated a stronger metastatic ability of cancer cells. Through common MAPK pathways, AA activated the p38 and ERK sub-pathways in a dose-dependent manner (Figure 6). This phenomenon was validated in patients with a history of traditional Chinese medicine use. These results may inform the development of alternative treatment plans for AA-related UTUCs.
